# Genetic Control of the Leaf Angle and Leaf Orientation Value as Revealed by Ultra-High Density Maps in Three Connected Maize Populations

**DOI:** 10.1371/journal.pone.0121624

**Published:** 2015-03-25

**Authors:** Chunhui Li, Yongxiang Li, Yunsu Shi, Yanchun Song, Dengfeng Zhang, Edward S. Buckler, Zhiwu Zhang, Tianyu Wang, Yu Li

**Affiliations:** 1 Institute of Crop Science, Chinese Academy of Agricultural Sciences, Beijing, 100081, China; 2 Institute for Genomic Diversity, Cornell University, Ithaca, New York, United States of America; Pennsylvania State University, UNITED STATES

## Abstract

Plant architecture is a key factor for high productivity maize because ideal plant architecture with an erect leaf angle and optimum leaf orientation value allow for more efficient light capture during photosynthesis and better wind circulation under dense planting conditions. To extend our understanding of the genetic mechanisms involved in leaf-related traits, three connected recombination inbred line (RIL) populations including 538 RILs were genotyped by genotyping-by-sequencing (GBS) method and phenotyped for the leaf angle and related traits in six environments. We conducted single population quantitative trait locus (QTL) mapping and joint linkage analysis based on high-density recombination bin maps constructed from GBS genotype data. A total of 45 QTLs with phenotypic effects ranging from 1.2% to 29.2% were detected for four leaf architecture traits by using joint linkage mapping across the three populations. All the QTLs identified for each trait could explain approximately 60% of the phenotypic variance. Four QTLs were located on small genomic regions where candidate genes were found. Genomic predictions from a genomic best linear unbiased prediction (GBLUP) model explained 45±9% to 68±8% of the variation in the remaining RILs for the four traits. These results extend our understanding of the genetics of leaf traits and can be used in genomic prediction to accelerate plant architecture improvement.

## Introduction

Over the past few decades, plant architecture improvement has greatly increased maize grain yields [1–3]. The leaves of maize hybrids in particular have become more upright. Erect leaves can effectively contribute to the maize grain yield by enhancing light capture for photosynthesis, serving as nitrogen reservoirs for grain filling and enabling denser planting with a higher leaf area index [4–6]. Therefore, understanding the genetic mechanisms of plant leaf architecture will not only address a fundamental issue in plant science but also facilitate the genetic improvement of maize breeding [7]. The leaf angle, leaf length, and leaf width are important components of maize leaf architecture. The leaf orientation value accounts for the ability of leaves to maintain the same orientation for their entire length, and it is a good selection index for plant leaf orientation [8].

Natural variations in leaf architecture have been identified by using quantitative trait loci (QTL) mapping in only a few different bi-parental maize mapping populations [9–13]. Mickelson et al. [9] found nine QTLs for the leaf angle by using a B73 x Mo17 population with 180 recombination inbred lines (RILs) and 192 genetic markers. Pelleschi et al. [10] detected five QTLs for leaf length and seven QTLs for leaf width under different water environments by using 120 RILs and 153 markers. Yu et al. [11] located a total of nine QTLs for the leaf angle in two different populations (120 F_2:3_ families with 102 SSR markers and 114 F_2:3_ families with 90 SSR markers). Lu et al. [12] located six QTLs for the leaf angle and eight QTLs for the leaf orientation value by using 397 F_2:4_ families and 137 SSR markers. Ku et al. [13] detected three QTLs for the leaf angle, three QTLs for the leaf length, four QTLs for the leaf width, and five QTLs for the leaf orientation value by using 229 F_2:3_ families and 222 SSR markers. The QTLs obtained through these studies usually had large confidence intervals, and thus it was difficult to identify the narrow recombination bins and underlying causal genes. Moreover, inconsistent results in term of the QTL location as found in the above studies had to be further validated. Tian et al. [14] used a nested association mapping (NAM) population in maize to conduct joint linkage mapping for the leaf architecture, resulting in 30 QTLs for the leaf angle, 36 QTLs for the leaf length, and 34 QTLs for the leaf width. Although 100 QTLs for leaf-related traits were obtained by Tian et al. [14], the parents of the NAM population only include a portion of the global maize diversity. Over approximately 500 years of selection, the Chinese maize germplasm has become well-adapted to the numerous ecological regions of China and is substantially different from US and Latin American germplasms [15]. Therefore, further research into the genetic mechanism underlying the leaf architecture could provide more favorable alleles for maize genetic improvement. In addition, when this NAM panel was studied, the RILs were only genotyped with 1106 SNP markers, which limited the high-resolution linkage QTL mapping.

High-throughput genotyping based on whole-genome sequencing data provides informative genome-wide and high density markers for mapping a population [16]. High-density markers can greatly improve the QTL mapping resolution and facilitate the identification of additional recombination events and exact recombination breakpoints. In rice, two different research groups used high-density genetic maps of two different RIL populations that were genotyped by sequencing to identify the QTLs, resulting in two QTLs for the grain length and width in regions of less than 200 kb that contained the *GS3* and *GW5* genes, respectively [17, 18]. In maize, an ultra-high density map of a large F_2_ population genotyped through GBS identified one QTL in a 700 kb region containing an *r1* gene that controlled the color of silk [19]. Therefore, the high-density map constructed by sequencing will be promising for candidate gene identification. Additionally, high-density genome-wide markers will lead to better genomic prediction in plant breeding. A genomic best linear unbiased prediction (GBLUP) that employs genomic relations to estimate the genetic merit of an individual has been shown to obtain accurate breeding values in breeding programs [20, 21]. This model allows for the prediction of breeding values in genotyped individuals before evaluating the phenotypic values of complex traits. This method will help breeders to select lines with superior potential performance from a larger germplasm pool and to accelerate genetic gains.

In this study, HUANGZAOSI was crossed as a common parent with three elite Chinese maize inbred lines to construct three connected RIL populations. These populations were used to conduct high-resolution QTL mapping for four leaf architecture traits within a single population and across all populations through GBS. A joint population analysis coupled with a high density genetic map provides high resolution for many QTL positions, permitting the robust evaluation of underlying candidate genes. Based on the genomic identity-by-state (IBS) matrices constructed through the GBS genotyping of the three populations, the cross-validated GBLUP model was applied to assess the accuracy of predicting each line’s mean trait value across the three populations.

## Materials and Methods

### Plant materials

Three recombination inbred line (RIL) populations were obtained by crossing the common parent HUANGZAOSI (HZS) with each of other three inbred lines, namely HUOBAI, WEIFENG322, and LV28. From the F_2_ progeny of each cross, a single seed descent was applied to produce RILs at the F_7_ generation. The HUOBAI, WEIFENG322, and LV28 populations included 183, 172, and 183 RILs, respectively. The parents of these populations were chosen on the basis of their different leaf architecture and maize germplasm groups. The common parent (HZS) is an important elite foundation inbred line with compact leaf architecture derived from Chinese Tangsipintou germplasm, a heterotic group used broadly in China. HZS was frequently used in Chinese maize breeding. In using HZSs as parental lines, more than 70 descended inbred lines and 80 important hybrids were released [22], with the total planting area of these hybrids exceeding more than 10 million ha 17 years ago [23]. HUOBAI and WEIFENG322 were two foundation inbred lines with semi-compact and expanded leaf architecture, respectively. LV28 is an elite foundation inbred line with expanded leaf architecture derived from Chinese Luda Red Cob germplasm, a heterotic group used broadly in China.

### Fields environments and trait evaluations

The phenotypes were measured in six field environments and performed over two years (2009 and 2010) at three different locations (Xinxiang of Henan province, Beijing, and Urumqi of Xinjiang province), where the institute of crop science belonging to the Chinese Academy of Agricultural Sciences has set up experimental field bases. The institute of crop science was approved for field experiments, and the field studies did not involve endangered or protected species. For each population, all lines were randomly grown within each replication with single-row plots of 11 plants. Two replications of each population were planted adjacent to one another. Each plot was 3 m in length and 0.6 m apart. Three representative plants from the middle of each plot were chosen to measure the four-leaf traits as follows at the 10^th^ day after anthesis. The leaf angle (LA) was scored as the angle of each leaf from a plane defined by the stalk below the node subtending the leaf. The leaf length (LL) was measured as the length from the base of the ligula to tip of the leaf. The leaf width (LW) was measured by taking the width of the widest section of the leaf. The leaf orientation value (LOV) was calculated as follows: LOV = 1/n ∑(90 − *θ*)×(*L*_*f*_ / *LL*) where *θ* is the leaf angle, *L*_*f*_ is the distance from the base of the ligule to the flagging point of the measured leaf, *LL* is the leaf length, and n is the number of measured leaves [13]. Three consecutive leaves including the first leaf above the primary ear, the primary ear leaf and the first leaf below the primary ear were measured for each of three plants. The trait value for each RIL was averaged for the three measured plants in each replication.

### Phenotypic data analysis

For each line of a single RIL population, the best linear unbiased prediction (BLUP) for all traits across environments were obtained by PROC MIXED in SAS 9.2. In the model, the environment, line, replication*environment and line*environment were considered as random effects. The broad-sense heritability (H^2^) for each trait across the environments was calculated on a plot basis by using the ANOVA tool in QTL IciMapping Version 3.3 [24]. The correlation coefficients among four traits were obtained on the basis of the BLUP with the *cor* function in the R software package [25].

### Genetic map construction and QTL mapping

The three RIL populations have been genotyped by using genotyping-by-sequencing (GBS) technology [26, 27]. The GBS data are available from S1 File. Recombination bin maps were constructed for each of the three populations, resulting in 1595, 1981, and 2091 bins for the HUOBAI, WEIFENG322, and LV28 populations, respectively. The composite recombination bin map including 4932 bins was also built across all the populations (unpublished). The bins were treated as genetic markers to construct a linkage map. The high-density maps had a powerful resolution for QTL mapping. The QTL analysis for the individual RIL population was conducted with inclusive composite interval mapping in QTL IciMapping software Version 3.3 [24]. The LOD threshold was determined by a 1000 permutation test. The *P* values for entering a variable (PIN) and removing a variable (POUT) were set at 0.001 and 0.002, and the scanning step was set to 1.0 cM. Joint linkage mapping across the three populations was conducted by PROC GLMSelect in SAS 9.2. The detailed mapping procedure was previously described [28]. The phenotypic variation explained (PVE) by each QTL was counted in a previously described study [29]. A 2 LOD-drop in the confidence interval was used for each QTL.

### Cross-validated genomic prediction across three RIL populations

The GBLUP of the rrBLUP package in R v3.0.2 was applied for genomic prediction [30]. We used the van Raden method [31] to construct the identity-by-state (IBS) genomic relationship matrix based on the GBS data of the three populations. All the RILs of the three populations were randomly divided into five disjointed subsets for cross-validation, where the line values from combinations of one to four subsets were used to calibrate models and predict the line values of remaining subsets [32]. This process was repeated 20 times. The prediction accuracy was counted as the coefficient of determination obtained by BLUP line means against the predicted line means obtained by GBLUP averaged over all the cross-validation runs.

## Results

### Phenotypic variation

**Fig 1:**
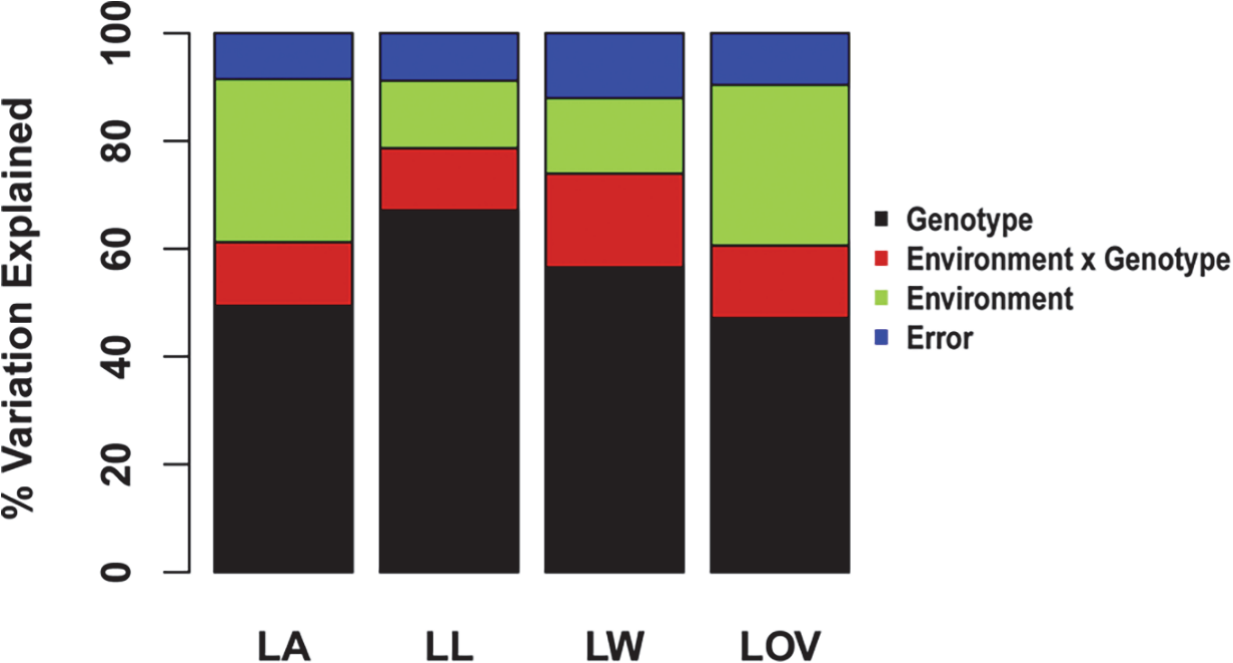
Partitioning variations in LA, LL, LW, and LOV across three populations.

Phenotypic variations were identified for LA, LL, LW, and LOV within three RIL populations (Table 1). The WEIFENG322 population had the greatest phenotypic variation for the four traits. The variation ranges for the four traits were similar between the HUOBAI and LV28 populations. The broad-sense heritability for LA, LL, LW, and LOV reached 0.68, 0.75, 0.63, and 0.64, respectively. Approximately 30.2% and 29.8% for LA and LOV variations across the three populations were attributed to environmental variations (Fig. 1). These variations were greater than that observed for LL or LW. Nonetheless, the manual phenotyping method for LA may confound measures of environmental variation.

**Fig 2:**
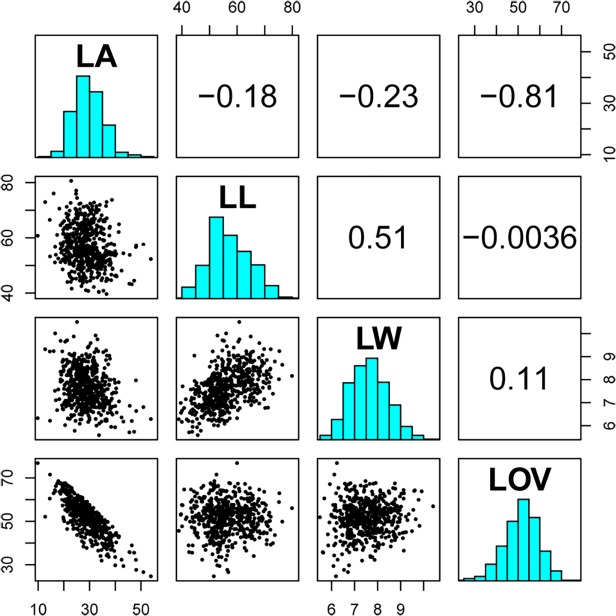
Trait correlations between LA, LL, LW, and LOV variations based on BLUP values across three populations. Right-top represents the correlation coefficients among four traits. Diagonal represents the frequency distribution for each of four traits. Left-bottom represents the scatter distribution among four traits.

The phenotypic correlations among the four traits are shown in Fig. 2. The largest correlation was estimated between LA and LOV (r = −0.81, p<0.0001). LA showed the most significant negative correlation to LL (r = −0.18, p<0.0001) and LW (r = −0.23, p<0.0001). The most significant positive correlation was observed between LL and LW (r = 0.51, p<0.0001).

**Table 1:** The mean, range, and difference within three populations and broad-sense heritability estimates (H^2^) across three populations for four traits.

Trait	Population	Mean	Range	Difference	H^2^
LA (degree)					0.68
	HUOBAI	28.6	15.4–47.2	31.8	
	WEIFENG322	31.2	10.6–54.6	44.0	
	LV28	28.9	19.1–42.3	23.2	
LL (cm)					0.75
	HUOBAI	52.1	39.6–67.4	27.7	
	WEIFENG322	54.2	40.4–76.1	35.6	
	LV28	64.7	53.7–80.6	27.0	
LW (cm)					0.63
	HUOBAI	7.1	6.0–8.7	2.7	
	WEIFENG322	7.6	5.6–10.5	4.9	
	LV28	8.2	6.6–9.7	3.2	
LOV					0.64
	HUOBAI	54.6	37.1–71.5	34.4	
	WEIFENG322	47.9	24.7–76.8	52.1	
	LV28	52.7	39.2–67.5	28.2	

### Linkage maps of recombination bins

Linkage maps for the HUOBAI, WEIFENG322, and LV28 populations were separately constructed by using 1595, 1981, and 2091 recombination bins, which were obtained through the genotyping-by-sequencing of the three RIL populations. The total lengths of the genetic linkage maps were 1746.61, 1960.57, and 1761.19 cM long with averages of 1.10, 0.99, and 0.84 cM between two neighboring bins for the HUOBAI, WEIFENG322, and LV28 populations, respectively. The composite map constructed from 4932 bins across the three populations was 1700.44 cM long with an average of 0.34 cM between two adjacent bins.

### Single population and combined multiple population QTL mapping

**Fig 3:**
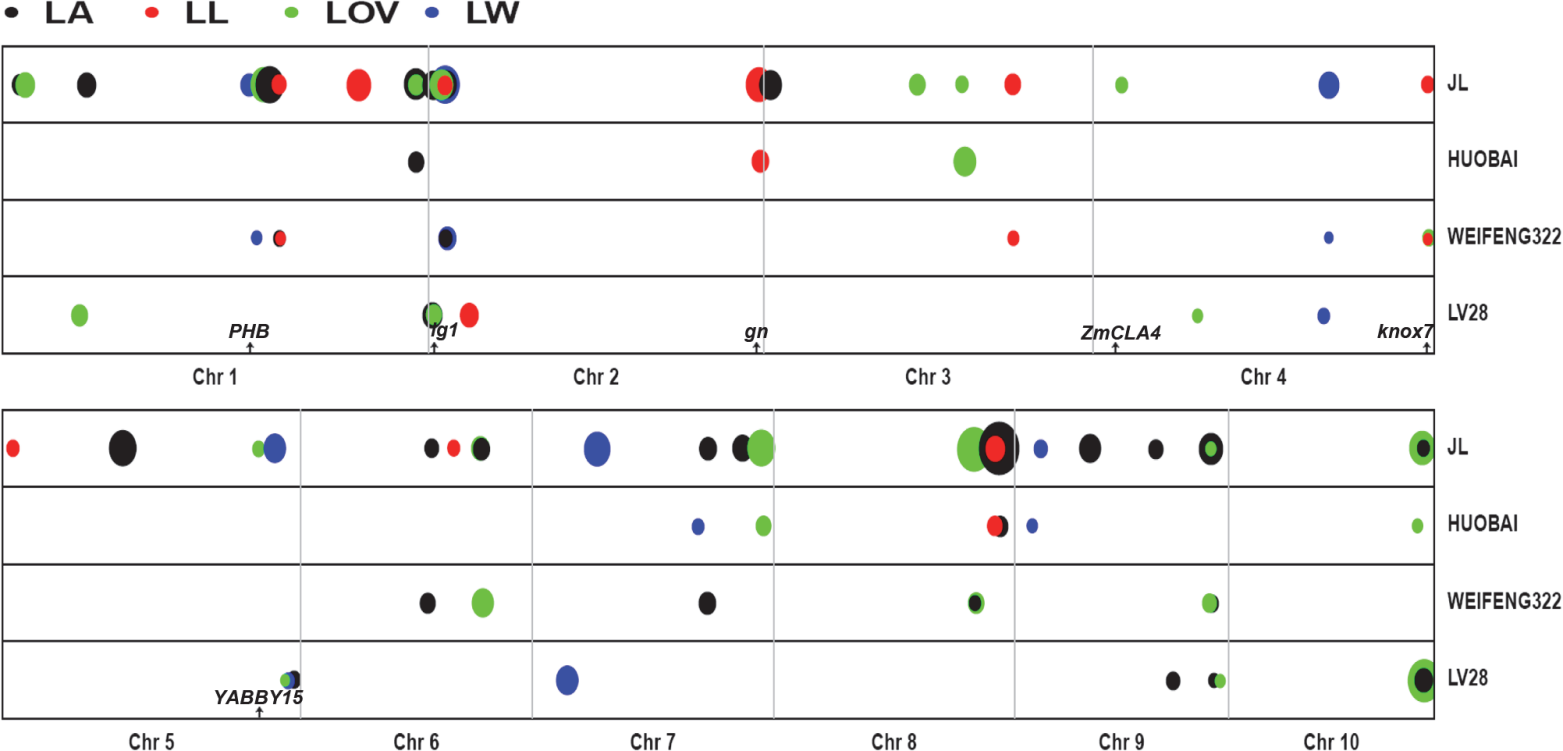
The leaf angle (LA), leaf length (LL), leaf width (LW), and leaf orientation value (LOV) QTLs obtained from single and joint-linkage QTL analyses across all the 10 chromosomes. Other parents were crossed with the HZS common parent to derive each bi-parental population as shown on the right vertical axis. The result of joint linkage mapping across the three populations is indicated as JL. The physical distance for each chromosome is represented in Mb units on the horizontal axis. Chromosomes were separated by solid gray lines. A solid circle size represents different LOD values ranging from 4.0 to 16.0. Different circle colors represent different traits. Arrows represent the physical location of the known maize leaf architecture genes overlapping with or adjacent to joint QTL.

A QTL analysis of each population was separately conducted for each trait, resulting in a total of 13, 6, 8, and 13 QTLs for the LA, LL, LW, and LOV, respectively (Fig. 3). We found that most of the QTLs for each trait were unique to a single mapping population. In general, the joint population analysis had much higher accuracy and power than single population mapping for detecting common QTLs [19]. Furthermore, joint linkage mapping across the three populations was performed to estimate the QTL position accurately for the four traits (Fig. 3, S1 Table). The joint analysis identified 17 QTLs for LA, 9 for LL, 6 for LW, and 13 for LOV, which collectively explained 58%, 59%, 51%, and 55% of the total phenotypic variations, respectively. The phenotypic variation explained (PVE) by individual QTLs across all populations ranged from 1.2% to 29.2% (S1 Table). A QTL for LL on chromosome 3 explained the largest proportion of phenotypic variance. The 2-LOD support interval of all QTLs mapped with the joint linkage analysis ranged from 0.602 Mb to 33.168 Mb, with a median of 2.838 Mb. The high resolution of the QTL position in the joint analysis greatly narrowed the genome distance in which causal genes could be searched through a forward genetic method.

The separate effect at each QTL for all three populations can be estimated by joint linkage analysis. A total of 34 alleles for LA, 14 for LL, 12 for LW, and 27 for LOV were significant at *P*<0.05 (S1 Table). All QTL additive allelic effects were small relative to the amount of variance observed among parental differences, with the largest allelic effects for LA, LL, LW, and LOV being 2.49°, 2.42 cm, 0.32 cm, and 2.67, respectively. The allelic series with both positive and negative additive allelic effects were identified in 28% to 61% of the QTL, depending on the trait.

### Candidate genes

With a relatively high mapping resolution, the small genomic regions of some QTLs can be used to search for candidate genes related to traits. For the leaf angle QTL, *qLA2a* was located in the region that ranged from 3.09 to 4.30 Mb on chromosome 2. The strong candidate gene *lg1* (*liguleless1*) that controls the leaf angle was previously cloned by mutagenesis in maize [33] and was located in this region. The *lg1* mutant has no ligule or auricle, leading to considerably more upright leaves than their normal counterparts. For the leaf length, *qLL2b* was mapped to the region that ranged from 233.55 to 236.61 Mb on chromosome 2, and the gene *gn* (*gnarley1*) that regulates leaf initiation and was cloned in maize [34] was found in that interval. *qLL4* is located in the 236.29–237.51 Mb region and was approximately 90 kb apart from the *knox7* (*knotted related homeobox 7*) gene, which regulated leaf initiation in maize [35]. For the leaf width, *qLW1* was overlapped with the position of the homolog for *Arabidopsis PHB*, which acts in the establishment of *Arabidopsis* leaf polarity [36]. For the leaf orientation value, the *qLOV4* spaced from 18.37 to 21.66 Mb on chromosome 4 was approximately 400 kb apart from the position of the *ZmCLA4* gene, which controlled the leaf angle and was cloned in maize [37]. *qLOV5* overlapped with the location of *YABBY15*, which was expressed on the adaxial side of the incipient and developing leaf primordia in maize [38].

### The genomic prediction of four traits

**Fig 4:**
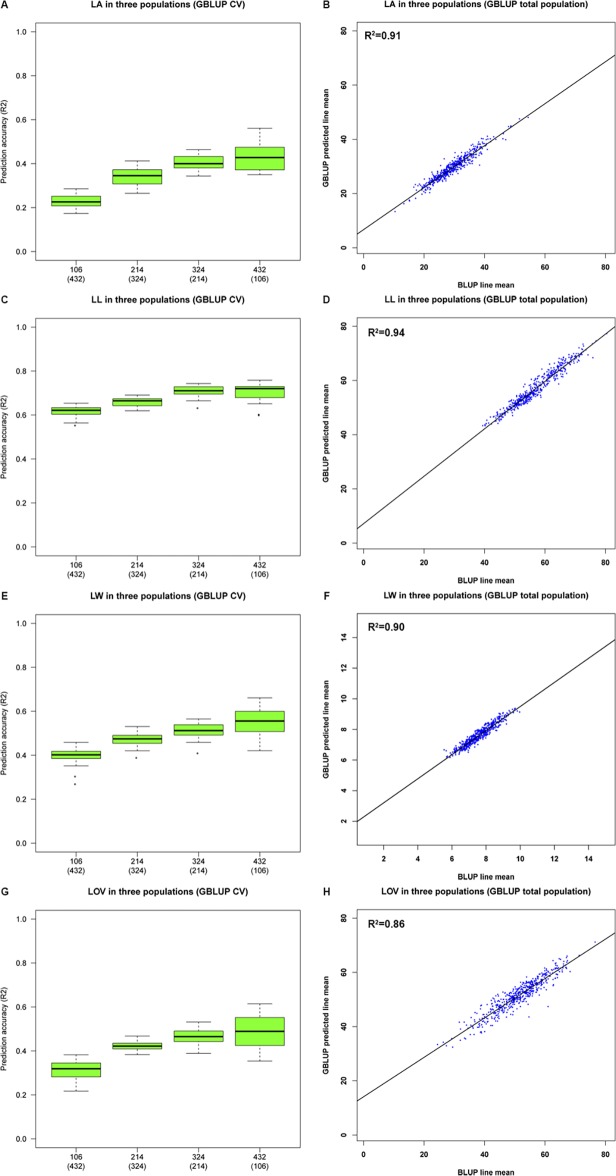
Genomic predictions of the leaf angle (LA), leaf length (LL), leaf width (LW), and leaf orientation value (LOV) in all RILs by GBLUP. A random sample of 20%, 40%, 60%, and 80% of the RILs in all populations for the calibrated GBLUP model to predict the BLUP line means variation in (A) LA, (C) LL, (E) LW, and (G) LOV in the remaining lines. All RILs in all populations were used to calibrate the GBLUP model to explain variations in (B) LA, (D) LL, (F) LW, and (H) LOV.

A genomic prediction by GBLUP was conducted to determine the ability of all genotyped diversity to explain variations in the BLUP line means for LA, LL, LW, and LOV across the three populations (Fig. 4). For the cross-validation of LA across all lines, 20% of the lines (106 RILs) that were randomly chosen from all the lines to calibrate a model could explain 23±5% of the LA variation in the remaining RILs (Fig. 4A). When 80% of the lines (432 RILs) were used to calibrate the model, the variation in LA that was explained increased to 45±9%. This finding suggested that the prediction accuracy of LA might be greatly improved as the calibration subset size increases. For LL variation, the prediction accuracy was strong in different calibration subset sizes (Fig. 4C). The calibration subsets of 106 RILs (20% of lines) explained 60±5% of LL variation in the remaining RILs. As the calibration subset size increased to 432 RILs (80% of lines), the prediction accuracy (68±8%) could not be substantially improved further. The LW cross-validation revealed that 20% of the RILs could explain only 37±10% of the LW variation in the remaining RILs (Fig. 4E). This percentage was much lower than the prediction accuracy of LL in the same calibration subset size. The prediction accuracy for LOV was a little more than that of the LA in different calibration subset sizes (Fig. 4A and 4G).

## Discussion

### Comparing the mapped QTLs and previously identified QTLs

We performed a literature review on the leaf architecture QTL as reported in maize in linkage mapping studies [9–14, 39] and compared the published QTL with those identified in this study. In the present study, fourteen of the 45 QTLs for leaf architecture could be validated by using the published QTL. For example, the known classic *lg1* gene controlling the leaf angle [33] was found to be located in an approximately 1-Mb region of *qLA2a* on chromosome 2. Tian et al. [14] also detected one significant QTL in the 2-Mb region at the nearby *lg1* on chromosome 2. Ku et al. [39] used a meta-QTL analysis to find an mQTL in the region between umc1165 and bnlg1297, which overlapped with the location of *lg1*. These QTLs that were detected in different genetic backgrounds and environments shared a high congruence, which strongly supported the candidacy of *lg1* for *qLA2a*. One QTL for LOV called *qLOV5* was identified on chromosome 5 in the region between 181.888 and 204.347 Mb. Mickelson et al. [9] and Lu et al. [12] also detected two QTLs for LA on chromosome 5, one QTL nearest to marker bnlg5.02 and the other one between umc1822 and phi048. Ku et al. [13] also found one important QTL for LA on chromosome 5 between bnlg1287 and mmc0282 and predicted *YABBY15* to be the candidate gene in this region. These previous results consistently suggested that this region contains a key gene for LA. Because the highest correlation occurred between LA and LOV, this region might include a gene that commonly controls the two traits. There was no such strong evidence for the remaining QTL in this study. However, we found that some candidate genes, such as *ZmCLA4* and *knox7*, perhaps support some of the detected QTLs.

In this research, we found thirty-one QTLs that were not reported in previous investigations. Several reasons may explain why these QTLs could be found in our current study. First, these QTLs were polymorphic in at least one population because QTL detection is generally based on natural allelic differences between parents [40, 41]. Second, the joint QTL mapping of multiple connected populations provides an improved power to detect QTLs [41–43]. For example, a joint linkage analysis of the three connected RIL populations could detect 17 QTLs for LA, 9 for LL, 6 for LW, and 13 for LOV; however, single RIL population mapping could only identify averages of 4, 2, 3, and 4 QTLs for LA, LL, LW, and LOV, respectively. Finally, high-density markers could increase the power of QTL mapping, especially for QTLs with small genetic effects [17]. Seven QTLs with PVE values >10%, namely *qLA9a*, *qLL1a*, *qLL3*, *qLW5*, *qLW7*, *qLOV3b* and *qLOV7*, were found in the thirty-one QTLs. *qLL1a*, *qLL3*, *qLOV3b* and *qLOV7* were still detected by individual population mapping. Although these major QTLs were not overlapped with the reported maize leaf architecture genes, they could be important target regions of fine mapping to identify candidate genes for leaf architecture, especially for the four major QTLs detected by joint linkage mapping and single population mapping. These new reported QTLs not only provide new genomic regions for the further identification and characterization of genes responsible for maize leaf architecture, but they also facilitate marker-assisted selection (MAS) for maize plant architecture improvement to develop hybrids that are better suited to high-density planting.

### Co-localized QTLs

A total of six co-localized QTL groups were detected among the 45 QTLs obtained by the joint linkage mapping for the four traits. Among these, the four groups located on chromosomes 1, 6, 9, and 10 were involved with the traits of both LA and LOV. To test the pleiotropy between LA and LOV, the correlation of allelic effect estimates across the three no-common parents at co-localized QTLs was found to be high (r = −0.61). Therefore, the same sets of genetic variants likely control natural variation at these co-localized loci between LA and LOV. The other two groups were identified as co-localizations between LW and LOV on chromosome 5, and among the LA, LL, and LW on chromosome 2. Few genetic overlaps were found among LA, LL, and LW in this study, which was consistent with the result obtained by Tian et al. [14]. Therefore, there were few pleiotropy locus functions among these traits.

### Predicting the leaf angle and related traits

Predicting the phenotypic value before trait evaluation could help breeders to optimize the allocation of evaluation resource to lines with the most predicted promise [44]. The GBLUP prediction model based on identity-by-state (IBS) has attracted interest in plant breeding [20, 21]. With the rapid progress of next-generation DNA sequencing technologies, millions of molecular markers could be used to estimate IBS genomic relations accurately [32]. To assess the prediction accuracies of our genetic models for LA and other traits, we evaluated the GBLUP performance across the three RIL populations. In cross-validations of the four traits, although the prediction accuracies of a given calibration set size were informative, variations in the prediction accuracies were present at all calibration set sizes. Variations in accuracy were relatively higher for LA and LOV than for LL and LW (Fig. 4). This finding may be attributed to the higher environmental variation for LA and LOV (Fig. 1). The GBLUP revealed significant and strong prediction accuracies for LL in different calibration set sizes (Fig. 4C). This trend suggested that new prediction models are needed to improve the prediction accuracy of LL. For LA, LW, and LOV, the prediction accuracies will largely depend on the calibration subset size. When all RILs were included in the calibration subset, variations in LA, LL, LW, and LOV explained by the IBS genomic relationship matrix were greater than 85% (Fig. 4). This finding revealed that substantial variations in the BLUP line means for LA, LL, LW, and LOV were predictable when using the GBLUP model. In conclusion, predicting the genetic value for leaf architecture can be an effective strategy for selecting lines whose phenotypes have yet to be observed in maize breeding.

## Supporting Information

S1 File Genotyping-by-sequencing (GBS) dataset for the HUOBAI, WEIFENG322, and LV28 populations. (ZIP)

S1 Table A summary of joint linkage analyses across the three RIL populations. (DOCX)
