# Supplementary material for: Genetic Control of the Leaf Angle and Leaf Orientation Value as Revealed by Ultra-High Density Maps in Three Connected Maize Populations
Source: PLoS One. 2015 Mar 25;10(3):e0121624. doi: 10.1371/journal.pone.0121624 (PMC4373667; doi:10.1371/journal.pone.0121624)
Supplement: S1 Table — (DOCX) [file pone.0121624.s002.docx]

S1 Table. A summary of joint linkage analyses across the three RIL populations.

| Trait | QTL | Chr | QTL peak and support interval physical position (Mb) | | PVE (%)^a^ | Additive effect^b^ | | |
| --- | --- | --- | --- | --- | --- | --- | --- | --- |
|  |  |  | QTL Peak | Support interval |  | HUOBAI | WEIFENG322 | LV28 |
| LA | qLA1a | 1 | 12.421352 | 10.842397-13.680845 | 2.0 | 1.09^*^ | 0.11^n^ | 0.35^n^ |
| LA | qLA1b | 1 | 59.389932 | 58.511395-63.203846 | 8.3 | -0.55^n^ | 1.56^*^ | -1.10^*^ |
| LA | qLA1c | 1 | 189.263675 | 188.109649-192.316788 | 6.7 | -0.88^*^ | -2.01^*^ | -0.89^*^ |
| LA | qLA1d | 1 | 292.427009 | 291.901726-292.875476 | 5.6 | -1.79^*^ | -0.25^n^ | -0.71^*^ |
| LA | qLA2a | 2 | 3.355919 | 3.095784-4.302914 | 4.3 | -0.71^*^ | -0.95^*^ | -1.47^*^ |
| LA | qLA2b | 2 | 12.106994 | 10.608170-12.798569 | 4.1 | 0.21^n^ | -1.49^*^ | -0.45^n^ |
| LA | qLA3 | 3 | 4.696962 | 4.146993-4.752983 | 3.7 | -0.99^*^ | -1.26^*^ | 0.01^n^ |
| LA | qLA5 | 5 | 88.125565 | 82.233998-90.426901 | 6.2 | 0.80^*^ | 1.97^*^ | 0.30^n^ |
| LA | qLA6a | 6 | 96.021155 | 91.986512-96.912270 | 2.7 | -0.55^n^ | -0.98^*^ | 0.44^n^ |
| LA | qLA6b | 6 | 132.353336 | 129.964080-132.795655 | 2.5 | 0.59^n^ | -0.80^*^ | -0.50^n^ |
| LA | qLA7a | 7 | 128.471794 | 126.222951-129.232426 | 4.6 | 1.07^*^ | 1.52^*^ | 0.21^n^ |
| LA | qLA7b | 7 | 153.985186 | 153.816711-155.252479 | 9.2 | -0.79^*^ | 1.85^*^ | -0.66^*^ |
| LA | qLA8 | 8 | 164.534564 | 164.035850-164.906982 | 7.6 | -1.96^*^ | -1.20^*^ | -0.23^n^ |
| LA | qLA9a | 9 | 55.569569 | 47.446929-77.912265 | 11.4 | 0.23^n^ | -2.49^*^ | 0.18^n^ |
| LA | qLA9b | 9 | 103.602333 | 102.440988-106.624963 | 8.6 | -0.20^n^ | 2.08^*^ | 1.10^*^ |
| LA | qLA9c | 9 | 143.486849 | 142.207738-144.612201 | 4.5 | -1.04^*^ | -1.37^*^ | -1.07^*^ |
| LA | qLA10 | 10 | 142.098792 | 141.649267-142.365313 | 6.9 | -0.88^*^ | 0.76^*^ | -1.51^*^ |
| LL | qLL1a | 1 | 195.447786 | 194.933070-196.692239 | 12.1 | -1.62^*^ | 1.20^*^ | 0.58^n^ |
| LL | qLL1b | 1 | 251.697274 | 250.888056-254.559362 | 9.3 | 0.89^*^ | 0.63^n^ | 2.07^*^ |
| LL | qLL2a | 2 | 11.716195 | 11.397789-13.542371 | 4.7 | -1.29^*^ | 0.85^*^ | -0.09^n^ |
| LL | qLL2b | 2 | 233.884554 | 233.550637-236.609537 | 4.9 | 1.11^*^ | 0.41^n^ | 1.25^*^ |
| LL | qLL3 | 3 | 176.379481 | 175.688605-179.123860 | 29.2 | -0.79^*^ | -2.09^*^ | 2.50^*^ |
| LL | qLL4 | 4 | 237.226121 | 236.297493-237.517527 | 6.0 | -0.53^n^ | -1.58^*^ | 0.45^n^ |
| LL | qLL5 | 5 | 7.465359 | 7.267703-7.982905 | 3.6 | 0.06^n^ | 0.44^n^ | 1.25^*^ |
| LL | qLL6 | 6 | 111.836369 | 107.88192-115.580615 | 1.2 | -0.53^n^ | -0.61^n^ | -0.27^n^ |
| LL | qLL8 | 8 | 161.528355 | 159.18925-161.634845 | 4.4 | 0.51^n^ | -0.73^n^ | 0.95^*^ |
| LW | qLW1 | 1 | 174.301468 | 172.470703-179.645151 | 5.3 | -0.07^n^ | 0.18^*^ | 0.07^n^ |
| LW | qLW2 | 2 | 12.246981 | 12.195485-13.296853 | 6.8 | 0.00^n^ | 0.20^*^ | 0.18^*^ |
| LW | qLW4 | 4 | 166.947991 | 165.993698-167.072246 | 7.6 | 0.05^n^ | 0.13^*^ | 0.25^*^ |
| LW | qLW5 | 5 | 199.246242 | 197.708917-202.132296 | 10.2 | -0.01^n^ | 0.18^*^ | 0.25^*^ |
| LW | qLW7 | 7 | 47.667594 | 41.423423-74.592334 | 12.2 | 0.13^*^ | 0.06^n^ | 0.32^*^ |
| LW | qLW9 | 9 | 19.089645 | 18.239308-23.275082 | 3.9 | -0.10^*^ | -0.16^*^ | -0.12^*^ |
| LOV | qLOV1a | 1 | 16.203723 | 15.907367-17.086260 | 2.8 | -1.46^*^ | -0.98^*^ | -0.91^*^ |
| LOV | qLOV1b | 1 | 184.665257 | 182.793128-187.398389 | 6.1 | 1.18^*^ | 1.49^*^ | 2.20^*^ |
| LOV | qLOV1c | 1 | 292.427009 | 291.725886-292.875476 | 9.5 | 2.12^*^ | -1.57^*^ | 0.75^n^ |
| LOV | qLOV2 | 2 | 9.106408 | 8.901876-9.503992 | 4.6 | 0.53^n^ | 1.08^*^ | 2.03^*^ |
| LOV | qLOV3a | 3 | 109.139482 | 88.946120-113.072881 | 6.5 | 0.51^n^ | 2.09^*^ | 1.92^*^ |
| LOV | qLOV3b | 3 | 140.478097 | 138.640916-145.476369 | 11.9 | 2.19^*^ | -1.83^*^ | -0.93^n^ |
| LOV | qLOV4 | 4 | 20.459184 | 18.370301-21.669671 | 4.3 | 1.17^*^ | -0.45^n^ | -1.26^*^ |
| LOV | qLOV5 | 5 | 186.983509 | 181.888644-204.347825 | 3.4 | -0.72^n^ | 1.27^*^ | 0.91^*^ |
| LOV | qLOV6 | 6 | 131.249272 | 130.130376-132.975209 | 4.9 | -0.48^n^ | 1.76^*^ | 1.21^*^ |
| LOV | qLOV7 | 7 | 167.386904 | 166.845534-168.166212 | 16.5 | 2.35^*^ | -2.67^*^ | -0.21^n^ |
| LOV | qLOV8 | 8 | 146.686607 | 146.152209-148.260467 | 7.2 | 1.43^*^ | 2.61^*^ | 0.49^n^ |
| LOV | qLOV9 | 9 | 143.486849 | 135.812894-145.059252 | 1.8 | 0.72^n^ | 1.29^*^ | 0.61^n^ |
| LOV | qLOV10 | 10 | 141.603436 | 141.543340-142.358868 | 7.6 | 1.77^*^ | 0.82^n^ | 2.44^*^ |

^a^ Phenotypic variation explained by each QTL.

^b^ Additive effect: positive values indicated that common parent HZS carries the allele for an increase in the traits, while negative values indicated that other parents contributed the allele for an increase in the trait value. * represented that allele effect is significant at P<0.05; n represented that allele effect is not significant at P<0.05.
